# Individual Pride and Collective Pride: Differences Between Chinese and American Corpora

**DOI:** 10.3389/fpsyg.2021.513779

**Published:** 2021-05-19

**Authors:** Conghui Liu, Jing Li, Chuansheng Chen, Hanlin Wu, Li Yuan, Guoliang Yu

**Affiliations:** ^1^Department of Psychology, Renmin University of China, Beijing, China; ^2^Department of Psychological Science, University of California, Irvine, Irvine, CA, United States; ^3^School of Education, Renmin University of China, Beijing, China

**Keywords:** corpora individual and collective pride, Chinese, American, collective pride, individual pride

## Abstract

This study investigated cross-cultural differences in individual pride and collective pride between Chinese and Americans using data from text corpora. We found higher absolute frequencies of pride items in the American corpus than in the Chinese corpus. Cross-cultural differences were found for relative frequencies of different types of pride, and some of them depended on the genre of the text corpora. For both blogs and news genres, Americans showed higher frequencies of individual pride items and lower frequencies of relational pride items than did their Chinese counterparts. Cross-cultural differences in national pride, however, depended on the genre: Chinese news genre included more national pride items than its American counterpart, but the opposite was true for the blog genre. We discuss the implications of these results in relation to the existing literature (based on surveys and laboratory-based experiments) on cultural differences in individual pride and collective pride.

## Introduction

Pride is a basic human emotion and refers to a self-conscious emotion derived from one's achievements. Depending on whether such achievements are attributable to oneself or to a group to which one belongs, the sense of pride is labeled as either individual or group/collective pride (Berkowitz and Levy, 1956; Zander et al., 1972; Chakrabarti, 1992; Liu et al., 2014). Examples of groups include one's family, friends, team, and nation. Previous research has documented cross-cultural differences in pride in general and in individual pride and collective pride in particular, but no study has examined cultural differences in individual pride and collective pride using text corpora.

A number of studies have shown that pride may be influenced by culture (Stipek et al., 1989; Mauro et al., 1992; Stipek, 1998; Eid and Diener, 2001; Scollon et al., 2004; Neumann et al., 2009). Eid and Diener (2001) identified the differences between individualistic cultures and collectivistic cultures with respect to self-reflective emotions (e.g., pride and guilt). Of relevance to the current study is the finding that the frequency and intensity of pride were lower in collectivistic cultures than in individualistic cultures. Because Eid and Diener (2001) used scenarios involving personal achievements, it is not known whether group or collective pride showed the same pattern of cultural differences. Recently, Sznycer and colleagues conducted two cross-cultural studies on pride-eliciting situations: one (Sznycer et al., 2017) involving 16 countries across four continents, but all being Western, Educated, Industrialized, and Democratic (WEIRD) cultures, and the other (Sznycer et al., 2018) involving 10 non-WEIRD small communities across Central and South Americas, Africa, and Asia. They found a high level of cross-cultural similarity (mean *r* = 0.81) within the WEIRD samples and a much lower level of agreement among the non-WEIRD samples (*r* = 0.21). It is also worth noting that like Eid and Diener (2001), Sznycer et al. (2017, 2018) focused on individual pride (all 10 scenarios in their 2018 study and 22 of the 25 scenarios in their 2017 study were about individual pride, with only three in their 2017 study being about relational pride; no scenarios covered national pride).

Theoretically, members of individualistic cultures tend to construe the self as separate from their social context and develop independent self-construal and individual pride, whereas members of collectivistic cultures tend to perceive the self as a constituent of the social context and thus develop interdependent self-construal and collective pride (Markus and Kitayama, 1991). Indeed, an earlier study indicated that different types of pride were distinctly influenced by cultural backgrounds. Stipek (1998) found that individuals in collectivistic cultures (e.g., China) would experience stronger pride if their children were accepted into a prestigious University than if they themselves were accepted, whereas people in individualistic cultures (e.g., the United States) would experience equal pride in these two circumstances. Through a cultural priming study, Neumann et al. (2009) also noted that cultural orientation affected the experience of pride: Interdependent self-construal priming led to greater pride in achievements of others, whereas independent self-construal priming led to greater pride in personal achievements.

Cross-cultural research on pride remains quite limited. Furthermore, relevant studies have primarily relied on the questionnaire or scenario data, which are subject to social desirability and self-serving biases (Taylor and Brown, 1988; Dunning et al., 1989, 1991; Lindeman and Verkasalo, 1995; Paulhus and John, 1998; Dunning, 1999; Robins and Beer, 2001). One method to overcome the limitations of self-report data is to use semantic analysis, which can mitigate ethnocentric bias (Gladkova, 2010) as well as social desirability and self-serving biases (Cohen, 2011). Several scholars have examined cultural differences using corpus-based methods (Gladkova, 2010; Apresjan, 2013). For example, Apresjan (2013) found that Russian speakers tended to express more passive emotions (e.g., fear) than English speakers, whereas English speakers tended to express more active negative emotions (e.g., anger).

Another limitation of the existing research on cultural differences in pride is its lack of differentiation of collective pride. Recent research has indicated that collective pride should be divided into relational pride and national pride (Liu et al., 2014; White and Branscombe, 2019). Relational pride concerns one's family, friends, and township (e.g., “When your friends achieve success” and “When your hometown is praised”). In contrast, national pride is tied to national achievements (e.g., “When a Chinese scientist receives the Nobel Prize”) (van Hilvoorde et al., 2010; Kavetsos, 2012; Meier and Mutz, 2016). Some studies have focused on national pride (Hjerm, 1998; Evans and Kelly, 2002; Smith and Kim, 2006), but they have not considered individual pride and relational pride.

The current study explored cultural differences in the frequencies of individual pride items and collective pride items between American and Chinese corpora. Considering that the suppression of pride is highly valued in China (Eid and Diener, 2001), we hypothesized that the absolute frequency of pride in the American corpus would be higher than that in the Chinese corpus. Within the pride items, however, we hypothesized that the frequency of mentions of individual pride would be higher in the American corpus than in the Chinese corpus, whereas the frequency of mentions of collective pride would be lower in the American corpus than in the Chinese corpus. Because previous research showed that emotional expressions differed by the genre of the corpus (Mahdar, 2018), we utilized two genres (blogs and news) to see whether cross-cultural differences depended on the genre.

## Method

### Corpus Selection

We used two freely available corpora in this study: the Corpus of Contemporary American English (COCA; available at https://www.english-corpora.org/coca/) and Beijing Language and the Culture University Corpus Center (BCC; available at http://bcc.blcu.edu.cn/). The COCA contains more than 1.0 billion words of the English text (20 million words per year from 1990 to 2019) and is equally divided among spoken language, fiction, magazines, newspapers, academic texts, web pages, blogs, and TV/movies. The spoken genre mainly included conversations from TV and radio programs in the United States, and the fiction genre included short stories and plays from magazines. Magazines and newspaper genres were from nearly 100 different magazines and newspapers from across the United States. The academic genre included literature from peer-reviewed journals covering the range of science, social sciences, and humanities. Web pages included web genres of academic, argument, fiction, information, and so on, from the US portion of the GLoWbE corpus. Blogs included texts from the US portion of the GloWbE corpus that were classified by Google as blogs. TV/movies included subtitles from OpenSubtitles.org and later the TV and movies corpora.

BCC contains nine languages, from which we selected modern Chinese. It includes more than 15 billion words of Chinese text (1990-2017) extracted from news, spoken language (e.g., microblogs), science and technology, literature, and other language styles. News genre materials were derived from the main newspapers in China, and the spoken genre mainly included information from microblogs. Science and technology genres included articles in academic journals published in China, and literature mainly included literary works.

To summarize, both corpora are very large and widely used and should be considered as comprehensive data of language use in their respective countries (Davies, 2010; Apresjan, 2013; Cheung and Larson, 2018; Darriba, 2019; Gao and Joh, 2019; Dang, 2020; Lu and Coxhead, 2020). To improve comparability of the two corpora, namely, blogs and news, we selected English blogs (only available for the year 2012) and Chinese blogs (only available for the year 2013). The news genres covered the years from 1990 to 2019 for both Chinese and English corpora.

### Sampling

In the English corpus, we selected “pride” and “proud” along with their grammatical variations, including “prided,” “prides,” “priding,” “prouder,” and “proudest” (Apresjan, 2013). In the Chinese corpus, we selected “自豪,” “自大,” and “骄傲,” which are Chinese synonyms for “pride” and “proud.” These words were used as search keywords. For each keyword found in the corpora, the search returned the text that included 20 Chinese characters or punctuations before each keyword and 20 Chinese characters or punctuations after each keyword in the Chinese corpus, and 15 words or punctuations on each side of the keyword in the English corpus. These strings of texts were considered as items. On occasions when multiple keywords were contained in the same item, they were treated either as a single occurrence when multiple occurrences were about the same type of pride or as separate occurrences when they were about different types of pride. We identified 10,909 and 10,542 items in the blog and news genres of COCA, respectively, and the corresponding numbers of items were 42,502 and 45,675 in the BCC. From these items, we randomly selected about 1,500 items from each type of items (1,533 English blog items, 1,462 English news items, 1,512 Chinese blog items, and 1,574 Chinese news items; the minor variations were due to an effort to ensure that the distribution of the various keywords in the final selection was the same as that for the corpora). In sum, we included 2,995 items from COCA and 3,086 items from BCC.

### Classification

Following Liu et al. (2014), we classified all items by pride type based on their linguistic context. Individual pride reflects personal qualities or behavior; relational pride concerns admirable qualities or behavior of one's friends, family members, hometown, or school; and national pride is related to admirable qualities or behavior of a nation (see Table 1).

**Table 1:** Examples of individual pride and collective pride.

**Type**	**English**	**Chinese**	
		**Original item**	**English translation**
Individual pride	No, I'm just joking. I've always **prided** myself on not having standard pick-up lines, which makes things that much more	不大愿意受人控制”、“你以 自己能独立思考而自 豪”、“你希望别人尊重 你”……诸如此类的描述	“[You] are not willing to be controlled by others,” “you are **proud** of your ability to think independently,” “you want others to respect you,” and so on
Relational pride	That women's chief priority is to serve their families, she not only took **pride** in her two daughters' achievements but also encouraged our career plans. We took	了!宝贝，加油!妈妈为你的 沉着和勇敢感到骄傲!但愿 大家都平平安安，和和美 美!但愿我的一	! Come on, Dear! Mom is **proud** of your composure and courage! May everyone be safe and at peace! I wish my
National pride	Must feel substantial and look majestic enough to engender national **pride**. # On the other hand, this currency cannot be costly to	愈来愈强盛。我不禁为祖国 的成就感到无比的自豪、骄 傲，在心中呼喊着：祖国， 生日快乐！我衷	More and more powerful. I cannot help but feel extremely **proud** of the achievements of my motherland, and I cry in my heart: Happy Birthday to my motherland! I sincerely
General pride	Physical gatherings and return to digital realms. Instead, alt-right gangs such as the **Proud** Boys, the Atomwaffen Division, and their followers, have become bolder, engaging in	大众音乐文化，倒真是“来 不得半点的虚伪和骄傲”。 听众一般并不会因为社会炒 作的厉害不厉害	The popular music culture, which really “cannot afford to have any dishonesty and **pride.”** The audience will generally not fall for any hypes
Non-Pride	Such as lions, which often hunt and raise their cubs along rivers. Lion **prides** have the highest reproductive success when their territories are in areas where rivers come together	好是个黄金单身汉 吧!12月21日综合报导自 大S替小S代班主持《康熙 来了》收视屡创新高	You'd better be a desirable bachelor! On December 21, it was reported that **since Big** S replaced Little S as the host, the TV show “Here Come Kang & Xi” has set a new record in its ratings. [“since big” has the same characters as self-pride in Chinese]

Four bilingual research assistants, one majoring in both psychology and English and the others majoring in psychology and passed College English Test Band 6 (CET-6, which is considered as proficient enough to read English newspaper articles and materials of general interests), were divided into two groups: One group classified 854 English and 1,589 Chinese items, and the other group classified 2,141 English and 1,497 Chinese items. These four coders were trained how to classify the text by type of pride: individual pride, relational pride, national pride, general pride (the items with little or no information to be coded by the above types of pride), and non-pride (the items with the keyword but not related to pride as an emotion, e.g., a pride of lions, see an example in Table 1). The two coders in each group practiced on 100 Chinese items and 100 English items and discussed any misclassifications. Finally, these two coders in each group independently coded all materials. For one group, the intercoder reliability (kappa coefficient) was 0.87 [95% CI: 0.85, 0.89] for the Chinese materials and 0.84 [95% CI: 0.81, 0.87] for the English materials. For the other group, the intercoder reliability was 0.90 [95% CI: 0.88, 0.92] for the Chinese materials and 0.82 [95% CI: 0.79, 0.84] for the English materials. A kappa coefficient of 0.81–1.00 is considered as showing as an almost-perfect agreement (Landis and Koch, 1977). For the items, the two coders disagreed (363 English items and 253 Chinese items); they were discussed and resolved in a group session involving the two coders as well as one of the corresponding authors. The non-pride items (170 English and 28 Chinese items) were excluded from further analysis, yielding 1,465 English items and 1,493 Chinese items from the blog genre and 1,360 English items and 1,565 Chinese items from the news genre in the final analysis.

The frequency results were analyzed using a chi-squared test. To determine which type of pride differed significantly between the two corpora, we calculated the adjusted standardized residuals (*z*-score) by standardizing the difference between observed values and expected values (Sharpe, 2015). The absolute value of adjusted residuals >2 is considered to be significant at α = 0.05 (Haberman, 1973).

## Results

The total frequencies of the pride items were much higher in the English corpus (86.93 and 85.74 usages per million words for the blog genre and the news genre, respectively), than in the Chinese corpus (14.17 and 22.84 usages per million words).

For the blog genre, a chi-squared test showed a significant association between culture (Chinese and American) and pride (individual, relational, national, and general), χ^2^(3, *N* = 2,958) = 97.13, *p* < 0.001, Cramer's *V* = 0.18, which indicates slightly larger than a medium effect size (0.17) according to Cohen (1988). For the news genre, a chi-squared test showed a significant association between culture and pride, χ^2^(3, *N* = 2,925) = 278.50, *p* < 0.001, Cramer's *V* = 0.31, which is larger than a large effect size (0.29) (Cohen, 1988). For both genres, compared with the Chinese corpus, the American corpus contained more items pertaining to individual pride and fewer items pertaining to relational pride (see Table 2). For national pride, cross-cultural differences showed opposite patterns for the two genres: The Chinese news genre contained more national pride items than its American counterpart, whereas the opposite was true for the blog genre. The remainder category (general pride) also showed opposite patterns—a higher frequency for Chinese blogs than for American blogs but a lower frequency for Chinese news than for American news.

**Table 2:** Individual pride and collective pride in Chinese and American corpora.

	**China**		**America**		***z-*score**
	**Frequency**	**Percent %**	**Frequency**	**Percent %**	
**Blog genres**					
Individual pride	547	36.6	660	45.1	−4.67
Relational pride	532	35.6	444	30.3	3.09
National pride	64	4.3	158	10.8	−6.73
General pride	350	23.4	203	13.9	6.75
**News genres**					
Individual pride	402	25.7	668	49.1	−13.40
Relational pride	657	42.0	430	31.6	5.84
National pride	414	26.5	117	8.6	13.23
General pride	92	5.9	145	10.7	−4.66

*The absolute value of z-score >2 is considered to be significant*.

## Discussion

We used the corpus-based methods to examine cultural differences in individual and collective (i.e., relational and national) pride between Americans and Chinese. These methods can mitigate to some extent the clear methodological disadvantages of questionnaire-based methods, including ethnocentric, social desirability, and self-serving biases (Taylor and Brown, 1988; Dunning et al., 1989, 1991; Lindeman and Verkasalo, 1995; Paulhus and John, 1998; Dunning, 1999; Robins and Beer, 2001; Cohen, 2011).

Our first finding was that the American corpus had higher baseline usage of pride-related words than the Chinese corpus. This finding suggests that Americans tend to express pride, whereas Chinese tend to suppress the expression of pride. Indeed, as mentioned earlier, Eid and Diener (2001) previously found that Chinese reported experiencing pride at a lower frequency and with a lower level of intensity than did Americans. More than for the emotion of pride, researchers (e.g., Russell and Yik, 1996) have recognized that Chinese people consider it to be a desirable trait to suppress many kinds of emotions (both positive and negative).

Within the usage of pride-related items, we found that the American corpus (of both news and blog genres) included a higher proportion of individual pride items and a lower proportion of relational pride items than the Chinese corpus. These findings are consistent with our hypothesis derived from a theoretical discussion of the implications of individualism-collectivism and independent/interdependent self-construal of emotions (Markus and Kitayama, 1991; Triandis and Gelfand, 1998; Brewer and Chen, 2007). Our results also echo empirical evidence from prior research using questionnaires (Stipek, 1998; Scollon et al., 2004) and cultural priming (Neumann et al., 2009).

Expressions of individual pride in interdependent cultures may be decreased or avoided to minimize potential interpersonal conflict and maintain social harmony (Scollon et al., 2004; Matsumoto et al., 2008). Indeed, individual pride may be associated with negative emotions in Asian cultures (Stipek, 1998; Scollon et al., 2004). Chinese individuals may also be more modest than individuals in Western cultures, especially in terms of individual pride (Heine et al., 1999). However, relational pride is encouraged in Eastern cultures; several studies have suggested that collective culture promotes pride for others (such as one's family, friends, and team) (Stipek et al., 1989; Neumann et al., 2009; Liu et al., 2014).

In terms of national pride, however, we found that cultural differences depended on the genre. The Chinese news genre contained more national pride items than did the American news genre, but the opposite was true for the blog genre. A possible explanation is that the traditional Chinese news media (such as newspapers and news journals) still play a significant propaganda-related role by focusing on the positive achievements of the country (Stockmann and Gallagher, 2011). In contrast, the American news media may be more critical of their government, hence focusing less on national achievements. Private citizens, on the other hand, might have reacted to the national news media and hence showed an opposite pattern. Future research is needed to test this speculation.

Finally, there were also cultural differences in the general pride category—more such items in the Chinese blog genre than in the American blog genre, but the opposite for the news genre. This pattern does not easily lend to a theoretically meaningful explanation. However, because these data were based on the proportions (i.e., relative to other categories), it might have just reflected the consequences of the other cultural differences discussed above. To our knowledge, this study is the first to examine cultural differences in individual and collective pride based on specific corpora. Our findings extend the collectivistic characterization of Chinese to include relational pride and national pride. The results also support the notion that culture as well as other factors (e.g., political factors) plays an important role in emotional expression (e.g., Mesquita and Frijda, 1992; Kitayama et al., 1997, 2006; Matsumoto et al., 1998; Mesquita, 2001; Tsai and Park, 2014; Tsai et al., 2016).

Several limitations of this study and their implications for future research need to be mentioned. First, we relied on limited cultural corpus samples (Chinese and American), to represent individualistic and collectivistic cultures. We did not include other major varieties of English language such as British and Australian English, which have been found to involve different cultural scripts in emotional expressions (Goddard, 2012). Future research should replicate our finding using corpora from other varieties of the English language as well as other languages representing individualistic cultures (e.g., German and French) and collectivistic cultures (e.g., Japanese, Korean, Mexican, and Spanish). Second, we did not analyze temporal changes in individual pride and collective pride. Several recent studies have indicated that cultural patterns can shift over time (Twenge et al., 2013; Hamamura and Xu, 2015; Zeng and Greenfield, 2015). Future research should consider time-series analysis. Such an approach may also reveal the importance of single events on national pride (c.f., the role of sporting events in national pride, Kavetsos, 2012). Finally, our text analysis can be complemented by cross-cultural experiments that would reveal relevant cognitive and emotional mechanisms.

## Data Availability Statement

Publicly available datasets were analyzed in this study. This data can be found at: http://bcc.blcu.edu.cn/; https://www.englishcorpora.org/coca/.

## Ethics Statement

This study was carried out in accordance with recommendations of the Research Ethics Committee of Renmin University of China with written informed consent from all subjects. All subjects gave written informed consent in accordance with the Declaration of Helsinki. The protocol was approved by the Research Ethics Committee of Renmin University of China.

## Author Contributions

CL and GY designed the original study and conducted final critical revision. JL, HW, and LY conducted literature searches and statistical analysis. CL and CC wrote and revised the manuscript. All authors contributed to the article and approved the submitted version.

## Conflict of Interest

The authors declare that the research was conducted in the absence of any commercial or financial relationships that could be construed as a potential conflict of interest.
